# Criterion Validity and Applicability of Motor Screening Instruments in Children Aged 5–6 Years: A Systematic Review

**DOI:** 10.3390/ijerph19020781

**Published:** 2022-01-11

**Authors:** Nienke H. van Dokkum, Sijmen A. Reijneveld, Judith Th. B. W. de Best, Marleen Hamoen, Sanne C. M. te Wierike, Arend F. Bos, Marlou L. A. de Kroon

**Affiliations:** 1 Department of Pediatrics, Division of Neonatology, Beatrix Children’s Hospital, University Medical Center Groningen, University of Groningen, Hanzeplein 1, 9713GZ Groningen, The Netherlands; n.h.van.dokkum@umcg.nl (N.H.v.D.); a.f.bos@umcg.nl (A.F.B.); 2 Department of Health Sciences, University Medical Center Groningen, University of Groningen, Hanzeplein 1, 9713GZ Groningen, The Netherlands; s.a.reijneveld@umcg.nl; 3 Department of Public Health, University Medical Center Rotterdam, Wytemaweg 80, 3015CN Rotterdam, The Netherlands; bfmtonderwijs@gmail.com (J.T.B.W.d.B.); hamoen.m@gmail.com (M.H.); 4 Center for Human Movement Sciences, University Medical Center Groningen, University of Groningen, Hanzeplein 1, 9713GZ Groningen, The Netherlands; s.c.m.te.wierike@umcg.nl; 5 Environment and Health, Department of Public Health and Primary Care, KU Leuven, Kapucijnenvoer 35 blok d, box 7001, B-3000 Leuven, Belgium; 6 Department of Obstetrics and Gynaecology, Erasmus MC—Sophia Children’s Hosptial, University Medical Center Rotterdam, Wytemaweg 80, 3015CN Rotterdam, The Netherlands

**Keywords:** screening, motor developmental problems, children, age 5–6, criterion validity

## Abstract

The detection of motor developmental problems, especially developmental coordination disorder, at age 5–6 contributes to early interventions. Here, we summarize evidence on (1) criterion validity of screening instruments for motor developmental problems at age 5–6, and (2) their applicability. We systematically searched seven databases for studies assessing criterion validity of these screening instruments using the M-ABC as reference standard. We applied COSMIN criteria for systematic reviews of screening instruments to describe the correlation between the tests and the M-ABC. We extracted information on correlation coefficients or area under the receiver operating curve, sensitivity and specificity, and applicability in practice. We included eleven studies, assessing eight instruments: three performance-based tests (MAND, MOT 4–6, BFMT) and five questionnaires (DCD-Q, PQ, ASQ-3, MOQ-T-FI, M-ABC-2-C). The quality of seven studies was fair, one was good, and three were excellent. Seven studies reported low correlation coefficients or AUC (<0.70), four did not report these. Sensitivities ranged from 21–87% and specificities from 50–96%, with the MOT4–6 having the highest sensitivity and specificity. The DCD-Q, PQ, ASQ-3, MOQ-T-FI, and M-ABC-2-C scored highest on applicability. In conclusion, none of the instruments were sufficiently valid for motor screening at age 5–6. More research is needed on screening instruments of motor delay at age 5–6.

## 1. Introduction

Motor developmental problems in children have a rather high prevalence, with several underlying causes. One of the most prevalent causes is development coordination disorder (DCD), with prevalence ranging from 5% to 15% [1]. Other causes of motor developmental problems are cerebral palsy and neuromuscular diseases [2], autism, attention deficit hyperactive disorder, intellectual and learning disabilities, and anxiety disorders [3]. Finally, an increasingly common cause of a motor developmental problem is a lack of opportunities to learn or practice motor skills in the home or school situation due to restrictive environmental factors [4].

Motor developmental problems can have detrimental consequences and its early detection and treatment can counteract these consequences. Detrimental consequences may be academic, emotional, and behavioral problems, such as anxiety, depression, low self-perception, and low self-perceived motor competence [5]. Motor developmental problems can also lead to an inactive lifestyle, thereby decreasing the level of physical fitness and increasing the risk of overweight [3,5,6,7]. These problems may in turn interfere with participation in play and sport, leading to further deterioration of motor skills. The prognosis of motor problems in children is rather poor [8,9,10], but timely interventions have been shown to improve motor performance and limit adverse consequences [11,12,13]. Early detection and treatment are thus warranted.

The early assessment of motor developmental problems is particularly relevant at the age of 5–6 years. It is the youngest age at which DCD can be diagnosed reliably based on its diagnostic criteria [1]. Moreover, at this age, motor developmental problems may become more urgent because these skills are necessary for participation in motor activities, such as sports. From this age onward, a positive school environment can provide opportunities to stimulate children with a motor developmental problem to prevent further deterioration. This is relevant for both the children with a motor developmental problem due to a biological or physical cause, and the children who have fallen behind because they have been understimulated at home. Cumulative effects may arise especially after this age, because of inducing a self-reinforcing cycle of understimulation and lower involvement in physical activities that increases the deviation from normal motor development at later ages [14].

Early detection of motor developmental problems requires a screening instrument with sufficient sensitivity and specificity, as well as practical applicability, that is validated by comparison to a standard diagnostic instrument. These standard instruments are usually motor performance-based tests, such as the Movement Assessment Battery for Children (first or second version, M-ABC(-2)) or the Bruininks–Oseretsky Test of Motor Proficiency-2 (BOT-2). Internationally, the M-ABC(-2) is the most commonly used performance-based diagnostic test in both clinical and research settings. Moreover, the M-ABC(-2) has been studied more extensively than the BOT-2 [3,15,16,17,18]. However, both tests are too time-consuming for routine early screening in the general population. Screening tests should have an adequate criterion validity (i.e., the degree to which the scores of screening instruments are an adequate reflection of a reference standard [19]) and be easily applicable in routine community-based practice, i.e., administration time and costs for materials and required training of professionals should be low. Unfortunately, a summary for the evidence on the criterion validity of screening instruments for motor developmental problems in children aged 5–6 years is lacking. Therefore, we conducted a systematic review to summarize (1) the evidence on the criterion validity of screening instruments for motor developmental problems in 5–6-year-olds with the M-ABC(-2) as reference, and (2) the applicability of these instruments in community-based settings.

## 2. Materials and Methods

To summarize the evidence on criterion validity, we followed the protocol for systematic reviews of measurement properties from Consensus-based Standards for the Selection of Health Measurement Instruments (COSMIN), [19] as described below. The review was registered in PROSPERO (ID 302069).

### 2.1. Study Selection

#### 2.1.1. Key Elements of the Research Question

Our research question had four key elements. First, the construct of interest, i.e., motor developmental problems. Second, the population of interest, i.e., children aged 5–6 years from a community-based population. Third, the type of measurement instrument of interest, i.e., all possible screening instruments to assess motor development, be it questionnaires or performance-based tests. Fourth, the measurement properties on which the review focuses, i.e., criterion validity and applicability.

#### 2.1.2. Search Strategy

The key elements of our research question were the basis of our search strategy. We systematically searched all published literature up to June 2021, using the following databases: Embase, MEDLINE (Ovid), Web of Science, PsycINFO-Ovid, CINAHL EBSCO, Cochrane, and Google Scholar. We used Endnote X9 (Clarivate, Philadelphia, PA, USA) for reference management.

#### 2.1.3. Eligibility Criteria

Studies were included if they met the following criteria: (1) the screening instrument was used to measure both gross and fine motor development; (2) the instrument was cross-sectionally compared to the M-ABC or M-ABC-2 test as reference standard; (3) the study reported or enabled us to determine (a) correlation coefficient or AUC, and (b) sensitivity and specificity; (4) the results were reported for children born in high-income countries (according to the definition by Statistics Netherlands [20]) aged 5–6 years and from a community-based population; and (5) the studies were published in English or Dutch. We excluded studies described in textbooks, studies with indirect evidence of measurement properties (such as randomized controlled trials), conference abstracts, and unpublished dissertations.

#### 2.1.4. Selection Procedure

Two authors (J.d.B., M.d.K.) applied the search strategy to all databases. Next, three authors (J.d.B., M.H., N.v.D.) independently screened titles and abstracts of studies for relevance. Discrepancies were managed by discussion to obtain consensus. In case of disagreement, the opinion of the last author (M.d.K.) was decisive, which happened in 4 papers which were not included. Full texts of eligible studies were retrieved and independently assessed by two of four authors (J.d.B., M.H., N.v.D., M.d.K.). Additionally, reference lists of the included studies were checked by one of three authors (J.d.B., M.H., N.v.D.) for relevance and potential inclusion.

### 2.2. Data Extraction

For data extraction, we used the form for validity studies as proposed by the COSMIN group, which includes descriptive characteristics of the study population, methods, and reported statistical findings of all investigated measurement properties [21]. We extracted information on two aspects of criterion validity, using the criteria proposed by Terwee et al. [22] and modified by Prinsen et al. [23]. The first aspect regards information on correlation coefficients or AUC. The second aspect concerns information on sensitivity and specificity, which is relevant for being able to study the effects on population level of screening tests [19,22,23]. For the data synthesis, we used the system originally developed as guideline for systematic reviews of trials in the Cochrane Collaboration Back Review Group [24] and adapted by Terwee et al. for use in systematic reviews of measurement properties [19]. Three authors (J.d.B., M.H., N.v.D.) independently applied the criteria for the findings reported in each study and for the data synthesis, as described under data analysis and reporting. In case of disagreement, discussion with the last author (M.d.K.) followed until consensus was obtained. To assess the applicability of the screening instruments, we obtained information on the screening instrument regarding (1) the number of items, (2) administration time, and (3) costs (i.e., demanded training for professional and material). This information was derived from the identified studies and other sources (i.e., manuals) if needed.

### 2.3. Quality Assessment of Studies

We assessed the quality of each screening instrument, using the COSMIN checklist developed by Terwee et al. [25]. This checklist includes items related to design, methods, and reporting. Each item can be rated with a 4-point scale from poor to excellent [25,26]. In Table 1, we show this checklist as used in our study. Three authors (J.d.B., M.H., N.v.D.) independently applied this checklist. In case of disagreement, discussion with the last author (M.d.K.) followed until consensus was reached.

### 2.4. Data Analysis and Reporting

First, we described the study flow and characteristics of the included studies. Second, we reported their methodological quality. Third, we summarized the criterion validity and applicability of all studies and addressed their overall suitability. We considered an instrument sufficiently suitable for screening purposes if the methodological quality of included studies was strong (Table 2) and the criterion validity was high (i.e., a correlation coefficient of the test with the criterion >0.70 as well as appropriate sensitivity and specificity for screening [19]). According to the norms of the American Psychological Association, a sensitivity of at least 80% and a specificity of at least 90% is preferable [27].

## 3. Results

### 3.1. Flow of Studies and Study Characteristics

**Figure 1:**
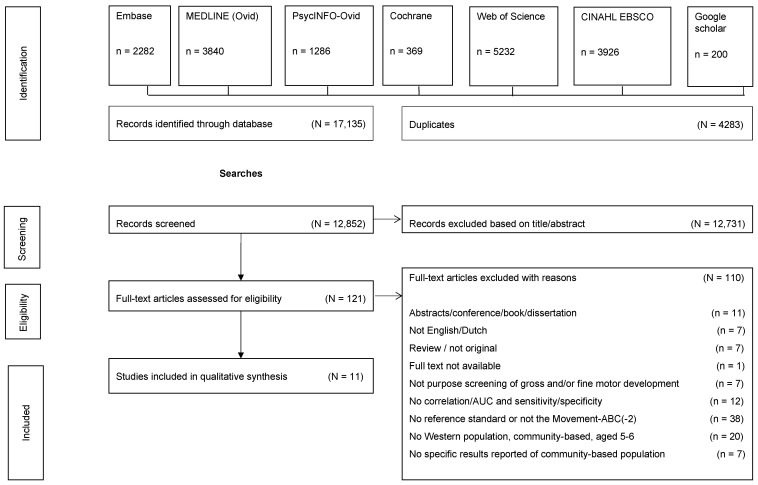
Flow of studies.

The flow of selection of papers is presented in Figure 1. We screened 17,135 studies, leading to an inclusion of 11 studies for data extraction, which assessed nine different screening instruments, fulfilling the inclusion criteria. One of the studies evaluated three different screening instruments, one of which was eligible for this review [28], and four studies evaluated the same instrument, the DCD-Q [29,30,31,32]. Screening instruments as studied regarded either tests or questionnaires (Table 3). Five regarded performance-based tests: the McCarron Assessment of Neuromuscular Development (MAND), the Motoriktest für vier- bis sechsjährige Kinder (MOT4–6, in English: Motor test for children aged 4–6 years), and the Baecke Fassaart Motor Test (BFMT). The other five screening instruments were questionnaires: the Developmental Coordination Disorder Questionnaire (DCD-Q); the Parental questionnaire (PQ); the Ages and Stages Questionnaire-3 (ASQ-3); the Motor Observation Questionnaire for Teachers, Finnish version (MOQ-T-FI); and the Movement Assessment Battery for Children-2 Checklist (M-ABC-2 Checklist or M-ABC-2-C). Reference standards regarded either the M-ABC test (six studies) [28,29,30,33,34,35] or the M-ABC-2 test (five studies) [31,32,36,37,38]. All studies made use of community-based samples; nine included these only [28,29,32,33,34,35,36,37,38] and the other two studies [30,31] combined community-based and selected samples. All studies were conducted in developed countries.

### 3.2. Quality of the Included Studies

The methodological quality was fair for seven studies (MAND [33], MOT 4–6 [34], DCD-Q [29,31,32], PQ [35], MOQ-T-FI [37]), good for the included test in one study (BFMT [28]), and excellent for three studies (DCD-Q [30], ASQ-3 [38], and M-ABC-2-C [36]). The fair ratings for methodological quality were mostly due to lack of clarity on the handling of missing data [28,31,32,33,34,35,37], small methodological flaws in design or execution [29,31,35], or a moderate sample size [34]. Less frequent methodological flaws concerned an unblinded M-ABC tester in the study on the DCD-Q [30], or not using a Danish M-ABC norm table in the study on the PQ [35].

### 3.3. Criterion Validity, Applicability, and Overall Suitability

In the following sections, we report on the key elements regarding criterion validity (in Table 4) and applicability (in Table 5) of included performance-based tests and questionnaires aimed at screening for motor developmental problems.

#### 3.3.1. Criterion Validity

In Table 4, we present that the criterion validity was poor for seven out of the eleven included studies because the correlation with the reference standard was smaller than 0.70 [28,30,33,34,36,37,38]. For the other four studies, the statistical findings were indeterminate [29,31,32,35]. In six out of eleven studies, the AUC was reported, ranging from 0.59 for the DCD-Q [29] to 0.91 for the SkSc-8 [28]. In Table 4, we also present that the sensitivities and specificities of the screening instruments varied widely: sensitivities ranged from 21% for the DCD-Q [31] and ASQ-3 [38] to 87% for the MOT4–6 [34] and specificities ranged from 50% for the MOQ-T-FI [37] to 96% for the ASQ-3 [38] at the applied cut-off points. Only the MOT4–6 met the American Psychology Association’s requirements of diagnostic accuracy based on the reported study results, with a sensitivity of 87% and a specificity of 90%, but had a correlation of <0.70 [34].

#### 3.3.2. Applicability

Regarding applicability, questionnaires scored better than performance-based tests (Table 5). The screening instruments largely differed with respect to number of items, administration time, and costs (i.e., demanded training for professionals and material). The number of items of the screening instruments varied from six (PQ) to thirty (ASQ-3 (with 12 questions about motor function). Administration time varied from about 10 min for the M-ABC-2-C and BFMT to about 25 min for the MAND. Noteworthy is that the DCD-Q and M-ABC-2-C provide information about the child’s participation in daily life, academics, and sport, while the other screening instruments do not. Regarding material costs, the MAND, MOT4–6, ASQ-3, M-ABC-2-C, and the BFMT had to be purchased, while the DCD-Q and the MOQ-T-FI were freely available (online). The PQ was used in the Danish National Birth Cohort Study and was freely available upon request from the authors.

## 4. Discussion

To our knowledge, our study offers the first summary of the evidence regarding criterion validity and applicability in community-based settings of screening instruments for motor developmental problems in children aged 5–6 years. We identified eleven relevant studies, in which nine different screening instruments were investigated that met our inclusion criteria. We found the validity of the identified instruments to be insufficient, being either poor [28,30,33,34,36,37,38] or indeterminate [29,31,32,35], with widely varying sensitivities and specificities. With respect to applicability, we found a large variation between the screening instruments. The overall quality of studies varied considerably from fair to excellent. From the data synthesis on the methodological quality and the statistical findings of the studies, ewe determined that none of the screening instruments have proven to be suitable for screening purposes.

We found criterion validity to be poor; none of the studies performed well. This may be partly due to the different balances between the subitems, representing different motor skills (e.g., fine versus gross motor skills) of the included screening instruments and the reference standard. For example, in the MAND, half (5 out of 10) of the tests are related to fine motor skills [33], whereas in the MOQ-T-FI, this only applies to 22% (4 out of 18) [36]. As in motor impairment, the deficits may vary between domains; a balance between the subitems in a screening instrument that differs from the reference standard may influence validity outcomes [39]. These findings underpin the conclusion of Fransen et al. [40], that different motor tests should be used depending on the specific aims, especially whether the focus should be either on gross or on fine motor skills. Differences in the share of fine and gross motor test items between tests and the M-ABC(-2) and differences in motor construct between the tests may account for the poor findings regarding criterion validity [28].

We found that the sensitivity and specificity at predefined current cut-off points varied widely, with the most favorable results for the performance-based tests. The better sensitivities and specificities of performance-based tests may be due to performance giving a more objective impression of the children’s skills than parent-reports on questionnaires, as has also been reported for language development in children [41]. Another explanation may be that no optimal cut-off points of the instruments have been chosen, as the reported AUCs of five instruments varied much less (i.e., from 0.59 to 0.67) than the sensitivities and specificities of these instruments (i.e., from 21% to 67%, and from 54% to 93%, respectively). For the BMFT, the optimal cut-offs were determined to obtain optimal sensitivity and specificity, rather than pre-set [28]. An optimization of the cut-offs of the various motor screening instruments for use in community-based settings should therefore be considered.

Overall, the MOT4–6 [33] and the BFMT [28] showed the most favorable measurement properties, i.e., rather high correlations and favorable balances between sensitivity and specificity (after optimizing the cut-offs of the BFMT), almost meeting the levels required for screening [27]. However, the quality of the only study on the MOT4–6 instrument was low (e.g., a very small sample size, n = 48), implying that the study results on the MOT4–6 must be considered with caution [34]. On the other hand, it should be noted that the defined criteria for validity, i.e., high specificity and corresponding sensitivity as proposed by the American Psychology Association [27] and high AUC or correlations of >0.70, may be too strict when assessing screening instruments for preventive child healthcare. Unlike population-based screening for diseases such as cancer, screening for motor developmental problems may be an ongoing process, with professionals following up on abnormal test scores with an extra consultation. Multiple consultations with repeated screening tests may enhance validity.

In summary, regarding sensitivity and specificity, the included performance-based tests perform better than the included questionnaires. Performance-based tests may provide a more objective view of motor developmental problems. Nonetheless, the evidence regarding screening with performance-based tests is also limited, so high-quality research is needed on these tests, especially the MOT4–6 and the BFMT, to further validate the results in population-based screening, and, additionally, to assess the added value of repeated measurements in clinical practice.

With respect to applicability, we found a large variation in relevant aspects of the screening instruments, such as administration time and costs (i.e., demanded training for professional and material). Performance-based tests generally scored worse on all aspects of applicability than questionnaires. This is largely due to performance-based tests requiring more administration time by a (trained) professional and test kits being more expensive than questionnaires. Performance-based motor screening tests may thus perform better than questionnaires, but at higher costs. However, one could also argue that performance-based tests have the disadvantage that they often only provide information at one time point, whereas parents or school teachers can monitor the child continuously.

Regarding the quality of studies, this was sufficient to high for six studies (BFMT [28], DCD-Q [30], ASQ-3 [38], M-ABC-2-C [36]). For these studies, results can be interpreted with a fair amount of certainty. For the other studies, the quality was low mostly due to lack of clarity in reporting or small methodological issues; results of these studies should be interpreted with caution. Future research on motor screening instruments should incorporate high-quality standards for all motor screening instruments on developmental problems. Future research may also incorporate populations from developing countries, as these underserved populations may have different economic, social, and educational conditions affecting a child’s development.

### Strengths and Limitations

Important strengths of this systematic review are that we systematically searched in a broad range of databases, that we used the COSMIN checklist to assess the methodological quality to study criterion validity, and that we used predefined criteria for rating the statistical findings. We further included both performance-based tests and questionnaires, enabling care-providers to make a well-considered choice between choosing one of these types of tests. A limitation of our study might be that we only searched for studies published in English and Dutch. We may have missed additional studies in other languages that met our other inclusion criteria, though these are likely rare. Finally, the COSMIN criteria have been suggested to be somewhat too strict, at the detriment of some medium-level evidence.

## 5. Conclusions

We conclude that the included studies provide insufficient evidence that the screening instruments are sufficiently valid as screening instruments for motor developmental problems in children aged 5–6 years. We therefore need better quality studies that may indicate needs for better quality screening instruments to timely identify motor developmental problems or the application of repeated measurements to realize sufficient sensitivity and specificity. Given the urgency of identification of motor developmental problems at age 5–6, especially DCD, we advise continued screening using the current best options.

## Figures and Tables

**Table 1:** COSMIN (Consensus-Based Standards for the Selection of Health Measurement Instruments) measurement property box for criterion validity [25].

	Excellent	Good	Fair	Poor
Was the percentage of missing items given?	Percentage of missing items described	Percentage of missing items NOT described		
2. Was there a description of how missing items were handled?	Described how missing items were handled	Not described but it can be deduced how missing items were handled	Not clear how missing items were handled	
3. Was the sample size included in the analysis adequate?	Adequate sample size (≥100)	Good sample size (50–99)	Moderate sample size (30–49)	Small sample size (<30)
4. Can the criterion used or employed be considered as a reasonable “reference standard”?	Criterion used can be considered an adequate “reference standard” (evidence provided)	No evidence provided, but assumable that the criterion used can be considered an adequate “reference standard”	Unclear whether the criterion used can be considered an adequate “reference standard”	Criterion used can NOT be considered an adequate “reference standard”
5. Were there any important flaws in the design or methods of the study?	No other important methodological flaws in the design or execution of the study		Other minor methodological flaws in the design or execution of the study	Other important methodological flaws in the design or execution of the study
*Statistical methods*				
6. For continuous scores: Were correlations or the area under the receiver operating curve calculated?	Correlations or AUC calculated			Correlations or AUC NOT calculated
7. For dichotomous scores: Were sensitivity and specificity determined?	Sensitivity and specificity calculated			Sensitivity and specificity NOT calculated

AUC = area under the ROC curve.

**Table 2:** Data synthesis based on (a) the methodological quality of the study and (b) the statistical evidence on the concurrent validity for measurement instruments, according to the COSMIN criteria [25,26].

Methodological Quality of Studies on One Instrument	Rating	Criteria
Strong	+++ or − − −	Consistent positive (+++) or negative (---) statistical findings in two or more studies of good methodological quality OR in one study of excellent methodological quality
Moderate	++ or − −	Consistent positive (++) or negative (--) statistical findings in two or more studies of fair methodological quality OR in one study of good methodological quality
Limited	+ or −	Positive or negative statistical finding in one study of fair methodological quality
Conflicting	+/−	Conflicting positive and negative findings
Unknown	?	Only studies of poor methodological quality

**Table 3:** Characteristics of the included studies.

Screening Instrument	Study	Ref. Std.	Setting and Population	Sample Characteristics	Test Protocol	Used Cut-Off
**Performance-based tests**						
MAND	Brantner et al. [33]	M-ABC	Australia, children aged 4 and 5 years recruited from regular schools/kindergartens. Children aged 6 years from another longitudinal study.	*n* = 118 (58 girls and 60 boys). Mean age 5 years and 1 month (SD = 8.28 months).	Assessment on MAND and M-ABC in random order, within one-week interval, individually by trained assessors.	M-ABC ≤ 15th percentile MAND NDI ≤ 85.
MOT4–6	Cools et al. [34]	M-ABC	Belgium (Flanders), children recruited from regular schools.	*n* = 48 (25 boys and 23 girls). Mean age 5 years and 6 months. (SD = 3 months).	Individual assessment with the MOT4–6 and the M-ABC with one-week interval by two trained examiners.	M-ABC ≤ 15th percentile MOT4–6 ≤ 16th percentile^.^
BFMT	De Kroon et al. [28]	M-ABC	The Netherlands, children aged 5 to 6 years, recruited from primary schools during physical education.	*n* = 116 (48 boys and 71 girls). Mean age 5.6 years (SD 0.28).	Individual assessment, both tests on the same day, performed by two assistants, trained in applying and scoring the tests according to protocol.	M-ABC ≤ 15th percentile reference population and own study population BFMT ≤ 10th percentile
**Questionnaires for parents**						
DCD-Q	Caravale et al. [29]	M-ABC	Italy, children recruited from regular schools.	Construct validity age 5–7 years *n* = 324 (173 boys and 151 girls). Test-retest reliability *n* = 45. Criterion age 5–7 years *n* = 19 (9 boys and 10 girls).	Parents filled in (1) a semi-open questionnaire to collect data about medical history and development, (2) the DCDQ-Italian (at home) and (3) consent for administration of the M-ABC if their child was selected. When indication or suspicion of DCD on the DCD-Q Italian, the M-ABC was administrated.	M-ABC ≤ 15th percentile DCD-Q ≤ 15th percentile
	Schoemaker et al. [30]	M-ABC	The Netherlands, children recruited from regular schools.	Age 4–8 years *n* = 182	Parents from children from the community-based population filled in the DCD-Q at home. M-ABC then administrated at school in 53% of the children (randomly selected), without prior knowledge of the children’s scores on the DCD-Q.	M-ABC ≤ 15th percentile DCD-Θ 15th percentile
	Kennedy- Behr et al. [31]	M-ABC-2	Germany, children recruited from regular preschools as part of another study.	*n* = 67 (32 boys and 35 girls). Mean age 66.51 months (SD = 4.41 months, range 60–75 months).	Validation of the final version of the DCDQ-G with community-based and selected population. Parents completed the DCDQ-G and children with a DCDQ-G total score of ≤ 49 were matched by age and gender with a child with total score ≥ 50 and tested with the M-ABC-2.	M-ABC 15th percentile, DCDQ-G total score < 47.
	Parmar et al. [32]	M-ABC-2	Canada, subset of children from a large, prospective cohort study, recruited from community-based organizations and Parent and Family Literacy Centers.	*n* = 181 (90 boys and 91 girls). Age 4–6 years. Mean age 5.01 years (SD = 0.79 months).	Parent filled in the DCD-Q’07.	M-ABC-2 ≤ 15th percentile, DCD-Q’07 total score < 46.
PQ	Nordbye-Nielsen et al. [33]	M-ABC	Denmark children recruited from Danish National Birth Cohort (DNBC).	*n* = 755 Mean age 5 years and 2 months (SD = 0.07).	Parents filled in 10 questions about motor function. The M-ABC was administrated by physiotherapists familiar with testing children.	M-ABC 5th percentile One question of the joint indicator (six questions) answered with yes.
ASQ-3	King-Dowling et al. [38]	M-ABC-2	Canada, subset of children from a larger cohort study, recruited from community-based organizations	*n* = 159 (76 boys and 83 girls). Mean age 53.1 months (range 43–65 months).	Parents filled in de ASQ-3 at home, 1 week prior the M-ABC-2 was administered. The Kaufman Brief Intelligence Test—Second Edition was also administered.	M-ABC-2 ≤ 16th percentile, ASQ-3 < 1.0 SD and < 2.0 SD, Kaufman Brief Intelligence Test-second edition ≤ 70
**Questionnaire for teachers**						
MOQ-T-FI	Asunta et al. [37]	M-ABC-2	Finland, recruited from regular pre-and elementary schools	Concurrent and predictive validity (Sample 1) age 6–12 years *n* = 193 (boys 48% and girls 52%). Mean age 9 years and 5 months. Construct validity and internal consistency (Sample 2) age 6–9 years *n* = 850 (boys 53% and girls 48%). Mean age 7 years and 7 months.	Two samples: Classroom teachers, physical education teachers, preschool teachers, special education teacher or other education professional filled in the web-based MOQ-T-FI. The M-ABC-2 was administered at school by trained physical education teachers.	M-ABC-2 not reported, MOQ-T-FI total score 37
**Questionnaire for parents or teachers**						
M-ABC-2-C	Schoemaker et al. [36]	M-ABC-2	The Netherlands and Belgium (Flandres), children were randomly selected by their teachers.	Age 5–6 years *n* = 191 (98 boys and 93 girls)	Teachers filled in the M-ABC-2-C and parents of randomly selected children filled in the DCD-Q’07 (n = 130). The M-ABC-2 was administrated by 16 therapists with at least four years’ experience.	M-ABC-2 and M-ABC-2-C ≤ 15th percentile.

MAND = McCarron Assessment of Neuromuscular Development; M-ABC = Movement Assessment Battery for Children; MOT4–6 = Motor Test for Children Aged 4–6 years; BFMT = Baecke Fassaart Motor Test; SkSc-8 = Skills Scan 8 items; SkSc-4 = Skills Scan 4 items; NDI = Neuro Development Index; SD = standard deviation; DCD = developmental coordination disorder; DCD-Q = Developmental Coordination Disorder Questionnaire; DCDQ-G = Developmental Coordination Disorder Questionnaire 2007 for German-Speaking Countries; PQ = Parental Questionnaire; ASQ-3 = Ages and Stages Questionnaire-3; MOQ-T-FI = Motor Observation Questionnaire for Teachers, Finnish version. Background indicates the category of instruments.

**Table 4:** Criterion validity of the screening instruments for detection of gross and/or fine motor developmental problems (adapted from Terwee et al.) [25].

Screening Instrument	Study	Methodological Quality	Statistical Rating	Data Synthesis	Sample Size (N)	Correlation * or AUC	Sensitivity (%); Specificity (%)
MAND	Brantner et al. [33]	Fair	-	Limited-	118	Correlation (M-ABC): *r* = −0.59 (*p* < 0.001) AUC: Not reported	72%; 80%
MOT4–6	Cools et al. [34]	Fair	-	Limited-	48	Correlation (M-ABC): - Total score: *r* *=* −0.68 (*p* < 0.01) - Gross motor score: *r* = −0.54 (*p* < 0.01) - Fine motor score: *r* *=* −0.29 (*p* < 0.05) AUC: Not reported	87%; 90% *
DCD-Q	Caravaleet al. [29]	Fair	?	Strong ---	324	Correlation: not reported AUC: 0.59	67%; 54%
DCD-Q	Schoemaker et al. [30]	Excellent	-		182	Correlation (M-ABC): - Total score 4–8 years: *r* = −0.24 (*p* = 0.001) AUC: Not reported	29%; 89%
DCD-Q	Kennedy -Behr et al. [31]	Fair	?		67	Correlation: Not reported AUC: 0.61	30%; 87%
DCD-Q	Parmar et al. [32]	Fair	?		181	Correlation: Not reported AUC: 0.66	21%; 93%
PQ	Nordbye-Nielsen et al. [35]	Fair	?	Limited-	755	Correlation: Not reported AUC: 0.61	40%; 88%
ASQ-3	King- Dowling et al. [38]	Excellent	-	Strong ---	159	Correlation (M-ABC-2): - Total score: *r* = 0.40 (*p* < 0.001) - Fine motor score: *r* = 0.31 (*p* < 0.001) - Gross motor score: *r* = 0.35 (*p* < 0.001) AUC: Not reported	Cut-off < 1SD 47%; 89% Cut-off < 2SD 21%; 96%
MOQ-T-FI	Asunta et al. [37]	Fair	-	Limited-	193	Correlation (M-ABC-2): - Total score: *r* = 0.37 (*p* < 0.001) AUC: Not reported	86%; 50%
M-ABC-2-C	Schoemaker et al. [36]	Excellent	-	Strong ---	191	Correlation (M-ABC): - Total score: *r* = −0.38 (*p* < 0.001) AUC: 0.67	41%; 88%
Baecke Fassaart Motor Test (BFMT)	De Kroon et al. [28]	Good	-	Limited+	116	Correlation (M-ABC): - Total score: *r* = −0.58 (*p* < 0.01) - Gross motor score: *r* = −0.63 (*p* < 0.01) - Fine motor score: *r* = −0.40 (*p* < 0.01) AUC: Cut-off 15th percentile of reference population: 0.85 Cut-off 15th percentile of study population: 0.87	Cut-off 15th percentile of reference population 79%; 78% Cut off 15th percentile of study population 78%; 81%

* Correlation: a positive or high (+) rating means that (1) the reported correlation coefficient with the reference standard is high (≥0.70) and (2) that there are convincing arguments that the reference standard is indeed a true reference standard (which is the case for the M-ABC(-2)). A negative (-) rating means that the reported correlation coefficient with the reference standard is low (<0.70) and/or the reference standard cannot be considered as gold. An indeterminate (?) rating means that there was no reported correlation with, or AUC related to, the reference standard. MAND = McCarron Assessment of Neuromuscular Development; MOT4–6 = Motor Test for Children Aged 4–6 years; BFMT = Baecke Fassaart Motor Test; SkSc-8 = Skills Scan 8 items; SkSc-4 = Skills Scan 4 items; DCD-Q = Developmental Coordination Disorder Questionnaire; PQ = Parental Questionnaire; ASQ-3 = Ages and Stages Questionnaire-3; MOQ-T-FI = Motor Observation Questionnaire for Teachers, Finnish version; M-ABC-2-C = Movement ABC-2 Checklist; M-ABC(-2) = Movement ABC(-2); FM = fine motor; GM = gross motor; AUC = area under the curve; * calculated by authors of this review.

**Table 5:** Applicability aspects of the screening instruments.

Screening Instrument	Age Group	Number of Items and Subscales	Response Options	Interpretation of Scores	Time to Administer and Training	Materials
**Performance-based tests**						
MAND [33]	3–16 years	5 fine motor tasks (one or two handed), 5 gross motor tasks(static and dynamic balance and postural control)	Time needed to perform the task	Neuromuscular Development Index	About 25 min and training needed for professional	Test kit
MOT4–6 [34]	4–6 years	18 items, four major performance areas: stability, locomotion, object control and fine movement skills	3 point rating scale and the possibility for qualitative notes about the performance	Total score with percentiles and Motor Quotients	About 15–20 min and training needed for professional	Test kit
BFMT [28]	5–6.5 years	13 items, gross motor (balance, locomotion and others) and fine motor skills	Sufficient motor control (1 point) or insufficient motor control (0 points)	Total score (maximum score = 13)	About 10 min per child and training needed for professional	Test kit
**Questionnaires for parents**						
DCD-Q [29,30,31,32]	5–14 years	15 questions, 6 questions about control during movement, 4 questions about fine motor activities and writing, 5 questions about general coordination	Likert scale	Total score with indication for DCD, or suspect, or probably no DCD	15 min, self-administered	Questionnaire and instruction form, freely available online
PQ [35]	5 years	158 questions, 6 domains (family and home, development, activity and friends, health, strengths and weaknesses, parents)	Dichotomous scale	Normal motor function or motor function delay	About 10 min, self-administered	Questionnaire available upon request to the author with the 10 questions about motor function (6 questions, are the “joint indicators”)
ASQ-3 [38]	1 month–5.5 years	30 items, in five areas with each 6 questions (communication, gross motor function, fine motor function, problem-solving, and personal-social)	Likert scale	Total score for each area	About 5 min, self-administered	Manual and questionnaire
**Questionnaire for teachers**						
MOQ-T-FI [37]	6–9 years	18 items, 14 questions about gross motor tasks, and 4 about handwriting/fine motor tasks	Likert scale	Total score. Higher scores reflect greater risk for motor problems	About 3.3 min, self-administered	Web-based questionnaire
**Questionnaire for parents or teachers**						
M-ABC-2 C [36]	5–11 years	30 items, two sections (movement in a static/predictable situation and in dynamic/unpredictable situation)	Likert scale	Total score of the two sections	About 10 min, self-administered	Manual and questionnaire

MAND = McCarron Assessment of Neuromuscular Development; MOT4–6 = Motor Test for Children Aged 4–6 years; BFMT = Baecke Fassaart Motor Test; SkSc-8 = Skills Scan 8 items; SkSc-4 = Skills Scan 4 items; DCD-Q = Developmental Coordination Disorder Questionnaire; PQ = Parental Questionnaire; ASQ-3 = Ages and Stages Questionnaire-3; MOQ-T-Fi = Motor Observation Questionnaire for Teachers, Finnish version; M-ABC-2-C = Movement Assessment Battery for Children-2 Checklist; DCD = developmental coordination disorder.

## Data Availability

No new data were created or analyzed in this study. Data sharing is not applicable to this article.

## Abbreviations

ASQ-3	Ages and Stages Questionnaire-3
AUC	Area Under the Receiver Operating Curve
COSMIN	Consensus-Based Standards for the Selection of Health Measurement Instruments
DCD-Q	Developmental Coordination Disorder Questionnaire
M-ABC(-2)	Movement Assessment Battery for Children (version 2)
M-ABC-2-C	Movement Assessment Battery for Children (version 2) Checklist
MAND	McCarron Assessment of Neuromuscular Development
MOQ-T-FI	Motor Observation Questionnaire for Teachers, Finnish version
MOT 4–6	Motoriktest für vier- bis sechsjährige Kinder (Motor Test for Children Aged 4–6 years)
BFMT	Baecke Fassaart Motor Test
PQ	Parental Questionnaire
